# Anesthetic Technique in a Patient With Multiple Sclerosis Scheduled for Laparoscopic Nephrectomy for a Renal Tumor: A Case Report

**DOI:** 10.5812/aapm.7550

**Published:** 2013-01-01

**Authors:** Arzu Acar, Mustafa Nuri Deniz, Elvan Erhan, Gulden Ugur

**Affiliations:** 1 Anaesthesiology and Reanimation Department, Ege University, Izmir, Turkey

**Keywords:** Multiple Sclerosis, Nephrectomy, Anesthesia, General, Prevention and Control

## Abstract

Perioperative stress and anesthesia are risk factors for exacerbation of Multiple Sclerosis (MS) attacks. Infection, emotional labiality and hyperpyrexia are also known to increase the risk of postoperative MS attacks. Appropriate preoperative evaluation, administration of a good premedication, control of fever, selection of the anesthetic agents and effective postoperative pain control can prevent problems after prolonged major surgery in patients with MS diagnosed. This report presents the anesthetic technique in a patient who was a known case of MS for past nine years and presented with renal tumor to undergo laparoscopic nephrectomy under general anesthesia.

## 1. Introduction

Multiple sclerosis (MS) is the most common immune demyelinating disorder of the central nervous system which predominantly affects females and play a major role which is involved in genetic factors (1). Perioperative stress and anesthesia are the factors that may have an effect on the exacerbation of MS attacks (1). Therefore, patients with MS undergoing anesthesia are at a higher risk of neurological dysfunction compared to those with normal neurological function (1). This case report presents the anesthetic technique in a patient with a diagnosis of MS who was planned to undergo laparoscopic nephrectomy for a renal tumor.

## 2. Case Report

A 57 years old female patient (weight 59 kg) who was a known case of MS for nine years presented with renal tumor to undergo laparoscopic nephrectomy. The patient’s history revealed that she initially had visual impairment and weakness of the legs which resolved with treatment. She has been using Copaxone (glatiramer acetate) therapy for six years and she had no attacks of MS during the last six years. Preanesthetic evaluation showed that she had no other medical diagnosis except MS. She had normal physical examination, PA chest radiograph, ECG and blood tests. She was premedicated with diazepam 5 mg orally the night before the operation. After routine monitoring and intravenous (iv) access were established, anesthetic induction was achieved with the intravenous administration of 60 mg of lidocaine, 180 mg of propofol, 100 μgr of remifentanil and 30 mg of atracuruim besylate. Anesthesia was maintained with O_2_-air-sevoflurane (2%). After intubation, a remifentanil infusion of 0.25 mcg/kg/min was administered. Body temperature ranged between 36.5 ºC and 35.7 ºC during the surgery. A total of 80 mg atracurium besylate was used as a neuromuscular blocking agent and the surgery lasted 7.5 hours. The dose of non-depolarizing muscle relaxant was chosen initially 0.5 mg/kg atracurium and repeated according to clinical signs and capnography. Two units of erythrocyte suspension were transfused during the surgery. After uneventful perioperative period. The patient was given meperidine via patient controlled analgesia (PCA) and intravenous paracetamol after every six hours during the postoperative 24 hours. The patient was discharged from the hospital 10 days later and had no MS attacks during the three-month follow up period.

## 3. Discussion

All anesthetic techniques used in patients with MS may lead to the exacerbation of MS symptoms. In a study by Barbosa et al. (2), subarachnoid anesthesia was performed with hyperbaric bupivacaine for a cesarean section in a pregnant woman with MS treated with methylprednisolone. No exacerbation of symptoms was noted in this patient. However, because the toxic effects of local anesthetics used for spinal anesthesia on demyelinated neurons may trigger MS attacks, MS is considered to be a relative contradiction to spinal anesthesia (1, 3). Furthermore, needle induced trauma and stress, the difficulty of the technique and hypotension during neuraxial anesthesia may lead to relapses in MS (3). Epidural anesthesia is considered to be safer than spinal anesthesia due to the lower prevalence of hypotension, and penetration of lower dose of local anesthetics into the intrathecal space (3). General anesthesia has its own advantages and disadvantages in patients with MS. There are studies reporting successful administration of inhalation anesthetics for the induction and maintenance of general anesthesia in patients with MS (1). We preferred general anesthesia in the case presented here because laparoscopic nephrectomy was planned for the removal of renal tumor. MS lesions may affect respiratory function by involving the inspiratory centers in the medulla oblongata or the cervical or thoracic spinal cord, motor function is usually reduced and even though total lung volume and vital capacity may be normal, maximal expiratory and inspiratory efforts may approach 50% of the normal values (1). Therefore, preoxygenation is important during induction of anesthesia. Additionally, medications used for the treatment of MS may interact with anesthetic agents, e.g., baclofen causes muscle weakness and produces an increased sensitivity to no depolarizing muscle relaxants (1, 4). In a study by Yamashita et al. (5), a patient with MS at exacerbation stage was monitored with processed electroencephalogram (pEEG) during anesthesia for aspiration curettage. In the mentioned case, anesthesia was induced with sevoflurane, N_2_O and O_2_. The authors concluded that, given that no spike was observed by pEEG monitoring during surgery, sevoflurane anesthesia was safe and can be used in the exacerbation stage in MS. In the case presented here, sevoflurane was used in the anesthetic procedure. In a study by Kono Y et al. (6) fentanyl. Propofol were used for induction, and intubation was carried out without the use of muscle relaxants and anesthesia was maintained with sevoflurane, O_2_ and air in a patient with MS undergoing emergency laparotomy and no postoperative exacerbation of the symptoms was observed. In a study by Inoue et al. (7), anesthesia was induced with propofol and fentanyl and maintained with sevoflurane, N_2_O and fentanyl, and vecuronium was used as neuromuscular blockers, and postoperative pain was managed with IV infusion of fentanyl instead of neural block with local anesthetics and no MS attacks were noted in a patient with a two year history of MS for gynecologic surgery. Lee KH et al. (8) reported a case report with a two year history of MS who was scheduled for emergency laparotomy. The authors also used propofol and remifentanil for induction of anesthesia and used sevoflurane and remifentanil for maintenance. The patient did not show postoperative exacerbation of MS symptoms. The authors point out the safety of sevoflurane for the anesthesia in this patient with MS. In accordance with these reports we also used sevoflurane and remifentanil infusion for the maintenance of anesthesia during laparoscopic nephrectomy which lasted 7.5 hours. We did not see exacerbation of MS symptoms in our patient postoperatively. Desflurane was also reported to be safe for MS patients in one study (9). However most of the studies report the use of sevoflurane for MS patients. Propofol infusion was also reported to be safe during anesthesia for MS patients (10). We used a non-depolarizing muscle relaxant atracurium (0.5 mg/kg initially followed by incremental doses) in our patient. It should be kept in mind that resistance to non-depolarizing neuromuscular blocking agents may develop due to increased number of acetylcholine receptors due to denervation (1, 11). On the other hand, since muscle weakness and decreased muscle mass are associated with increased sensitivity to neuromuscular blocking agents, titration, continual monitoring and use of the lowest dose are required. Operative stress and anesthesia may exacerbate the symptoms of MS. Spinal anesthesia, because it exacerbates MS attacks, is a relative contraindication. General anesthesia and epidural anesthesia, which allow the use of low dose local anesthetics, are safer. Since increased body temperature may lead to relapses in patients with MS (1, 12) perioperative and postoperative temperature monitoring is very crucial for the perioperative management of patients with MS. In conclusion, appropriate evaluation and selection of the anesthetic agents during the perioperative period, perioperative fever control and effective postoperative pain control were important factors for prevention of MS related problems in this patient.
